# Complete mitochondrial genome sequence of *Cicindela anchoralis* Chevrolat, 1845 (Coleoptera: Carabidae)

**DOI:** 10.1080/23802359.2018.1443040

**Published:** 2018-02-24

**Authors:** Ah Rha Wang, Min Jee Kim, Su Yeon Jeong, Iksoo Kim

**Affiliations:** Department of Applied Biology, College of Agriculture & Life Sciences, Chonnam National University, Gwangju, Republic of Korea

**Keywords:** Mitochondrial genome, Carabidae, *Cicindela anchoralis*, Endangered species, phylogeny

## Abstract

The tiger beetle, *Cicindela anchoralis* Chevrolat, 1845 (Coleoptera: Carabidae), has been listed as an Endangered insect in South Korea. We sequenced the complete mitochondrial genome (mitogenome) of this organism (16,388 bp). The genome includes a typical set of genes (13 protein-coding genes (PCGs), 2 rRNA genes, and 22 tRNA genes) and 1 non-coding region with an arrangement identical to that observed in most insect genomes. Twelve PCGs had the typical ATN start codon, whereas *ND1* had the atypical TTG codon. The AT-rich region is 1629-bp long, composed of 80.0% A + T nucleotides, and has no long repeat sequences. Phylogenetic analyses with concatenated sequences of the 13 PCGs and 2 rRNA genes, using the Bayesian inference (BI) method, placed *C. anchoralis* as a sister to the within-subfamilial species *Habrodera capensis*, with the highest nodal support presented by both BI and maximum likelihood (ML) methods. Three subfamilies represented by more than one species (Cicindelinae, Harpalinae, and Carabinae) were all determined by both BI and ML analyses to form strong monophyletic groups.

The tiger beetle, *Cicindela anchoralis* Chevrolat, 1845 (Coleoptera: Carabidae), which has been listed as an Endangered insect in South Korea, is distributed throughout South Korea, Japan, Taiwan, and China (Kim 2003). In Korea, the species occurs on the sandy beaches of the western coastline during July to August (Shin et al. 2013).

An adult male *C. anchoralis* was collected from Taean-gun, Chungcheongnam-do Province (36° 36′ 52.00″ N, 126° 17′ 17.62″ E), South Korea, in 2017. This voucher specimen was deposited at the Chonnam National University, Gwangju, Korea, under the accession no. CNU7047. Using DNA extracted from the hind legs, two long overlapping fragments (LFs; *COI*-*CytB* and *CytB*-*COI*) were amplified using two sets of primers designed from the available mitogenomes of Coleoptera (Song et al. 2010; Wan et al. 2012; Kim et al. 2014; Linard et al. 2016; López-López and Vogler 2017). Subsequently, these LFs were used as templates to amplify 30 short fragments. The sequence data has been deposited in GenBank under the accession number MG253029.

We performed phylogenetic analysis using the concatenated nucleotide sequences of 13 protein-coding genes (PCGs) and two rRNA genes of 16 mitogenome sequences from Carabidae in Coleoptera. Bayesian inference (BI) and maximum-likelihood (ML) methods that were implemented in CIPRES Portal v. 3.1 (Miller et al. 2010) were used for phylogenetic analyses. An optimal partitioning scheme (six partitions) and substitution model (GTR + Gamma + I) were determined using PartitionFinder 2 and the Greedy algorithm (Lanfear et al. 2012, 2014, 2016).

The complete 16,388-bp mitogenome of *C. anchoralis* was composed of 2 rRNAs, 22 tRNAs, 13 PCGs, and 1 major non-coding region referred to as the A + T-rich region (1629 bp). The arrangement of this genome was identical to that typically observed for other insects (Cameron 2014). The A + T content of the whole mitogenome was 73.8%; however, it varied among the genes as follows: AT-rich region, 80.0%; lrRNAs, 79.4%; srRNAs, 77.0% and PCGs, 71.5%. Twelve PCGs had the typical ATN start codon, whereas *ND1* had the atypical TTG codon. Ten of the 13 PCGs had a complete stop codon (six TAA and four TAG); however, *COII*, *ND5*, and *ND4* had an incomplete stop codon, T or TA. Unlike other coleopteran AT-rich regions, which have long AT-rich regions (e.g. 4469 bp in *Coccinella septempunctata* (Kim et al. 2012); 5654 bp in *Protaetia brevitarsis* (Kim et al. 2014)) the 1629-bp-long AT-rich region of *C. anchoralis* does not harbour any long repeat sequences; however, it does harbour several AAATTTT sequences, and has multiple runs of TA sequences scattered throughout.

Results of the phylogenetic analysis indicated a sister relationship between *C. anchoralis* and the within-subfamilial species *Habrodera capensis,* with the highest nodal support by both BI and ML methods (Bayesian posterior probabilities [BPP] = 1; Bootstrap [BS] = 100; Figure 1). The three subfamilies in Carabidae represented by more than one species all formed strong monophyletic groups (BPP = 1; BS = 100; Figure 1). Currently, only a limited number of complete mitogenome sequences are available from this family. Therefore, more complete mitogenome sequences are required from this family in order to obtain data compatible with those obtained using other molecular markers.

**Figure 1.**
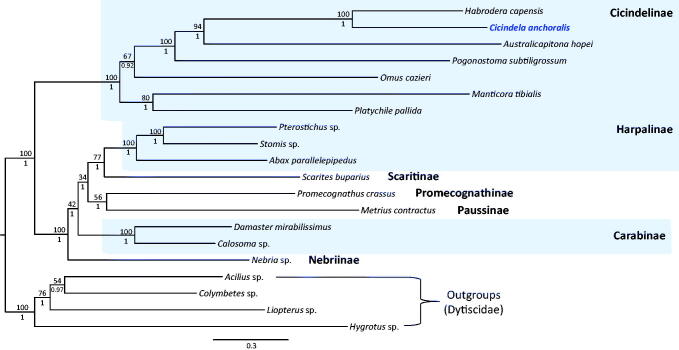
Bayesian inference (BI) method-based phylogenetic tree constructed for the family Carabidae using the concatenated sequences of 13 PCGs and two rRNA genes. The numbers at each node indicate the bootstrap support using the maximum likelihood (ML) method (above nodes) and the Bayesian posterior probabilities (BPP; below nodes), using the BI method. The scale bar indicates the number of substitutions per site. Four species belonging to the family Dytiscidae were used as outgroups. GenBank accession numbers are as follows: *Habrodera capensis*, JX412824 (Timmermans et al. 2015); *Australicapitona hopei*, MF497816 (López-López and Vogler 2017); *Pogonostoma subtiligrossum*, MF497820 (López-López and Vogler 2017); *Omus cazieri*, MF497813 (López-López and Vogler 2017); *Manticora tibialis*, MF497821 (López-López and Vogler 2017); *Platychile pallida*, MF497814 (López-López and Vogler 2017); *Pterostichus* sp., KT876909 (Linard et al. 2016); *Stomis* sp., KT876914 (Linard et al. 2016); *Abax parallelepipedus*, KT876877 (Linard et al. 2016); *Scarites buparius*, MF497821 (López-López and Vogler 2017); *Promecognathus crassus*, JX313665 (Timmermans et al. 2015); *Metrius contractus*, MF497817 (López-López and Vogler 2017); *Damaster mirabilissimus*, GQ344500 (Wan et al. 2012); *Calosoma* sp., GU176340 (Song et al. 2010); *Nebria* sp., KT876906 (Linard et al. 2016); *Acilius* sp., KT876878 (Linard et al. 2016); *Colymbetes* sp., KT876885 (Linard et al. 2016); *Liopterus* sp., KT876902 (Linard et al. 2016) and *Hygrotus* sp., KM244659 (Timmermans et al. 2015).
